# Evaluating Clinical Educators' Competence in an East Asian Context: Who Values What?

**DOI:** 10.3389/fmed.2022.896822

**Published:** 2022-06-28

**Authors:** Chang-Chyi Jenq, Liang-Shiou Ou, Hsu-Min Tseng, Ya-Ping Chao, Jiun-Ren Lin, Lynn V. Monrouxe

**Affiliations:** ^1^Department of Nephrology, Linkou Chang Gung Memorial Hospital, Taoyuan, Taiwan; ^2^Chang Gung University College of Medicine, Taoyuan, Taiwan; ^3^Chang Gung Medical Education Research Center, Taoyuan, Taiwan; ^4^Division of Allergy, Asthma and Rheumatology, Department of Pediatrics, Chang Gung Memorial Hospital, Taoyuan, Taiwan; ^5^Department of Health Care Management, College of Management, Chang Gung University, Taoyuan, Taiwan; ^6^Faculty of Medicine and Health, The University of Sydney, Sydney, NSW, Australia

**Keywords:** multisource feedback, faculty assessment, faculty development, clinical educator, multisource evaluation

## Abstract

**Background:**

*How* to evaluate clinical educators is an important question in faculty development. The issue of *who* are best placed to evaluate their performance is also critical. However, the *whos* and the *hows* of clinical educator evaluation may differ culturally. This study aims to understand what comprises suitable evaluation criteria, alongside who is best placed to undertake the evaluation of clinical educators in medicine within an East Asian culture: specifically Taiwan.

**Methods:**

An 84-item web-based questionnaire was created based on a literature review and medical educational experts' opinions focusing on potential raters (i.e., *who*) and domains (i.e., *what*) for evaluating clinical educators. Using purposive sampling, we sent 500 questionnaires to clinical educators, residents, Post-Graduate Year Trainees (PGYs), Year-4~6/Year-7 medical students (M4~6/M7) and nurses.

**Results:**

We received 258 respondents with 52% response rate. All groups, except nurses, chose “teaching ability” as the most important domain. This contrasts with research from Western contexts that highlights role modeling, leadership and enthusiasm. The clinical educators and nurses have the same choices of the top five items in the “personal qualities” domain, but different choices in “assessment ability” and “curriculum planning” domains. The best fit rater groups for evaluating clinical educators were educators themselves and PGYs.

**Conclusions:**

There may well be specific suitable domains and populations for evaluating clinical educators' competence in East Asian culture contexts. Further research in these contexts is required to examine the reach of these findings.

## Introduction

Teaching effectiveness and faculty development are central to healthcare professional excellence (1, 2). Indeed, clinical educators are asked to play numerous roles in relation to their students. For example, Harden and Crosby identify twelve roles of clinical teachers in medical education accross six areas: the *information provider* in the lecture, the *role model* on the clinical job, the facilitator as a *mentor*, the *assessor* of learners, the *curriculum planner*, and the *resource developer* (3). Thus, in medical education, clinical educators focus not only on educating students in terms of their medical knowledge, but also on the learners' learning state, living conditions and emotional state. Indeed, such psychosocial support includes counseling, friendship, acceptance, confirmation and role-modeling (4, 5). Due to the multiple functions of clinical educators, adequate evaluation of their performance is a challenging and complex task.

### What Competencies Need to Be Evaluated in Our Clinical Educators?

In 2010, a systemic review of over 30 years' research of current questionnaires for evaluating clinical educators identified 54 manuscripts focusing on 32 different instruments from predominately Western (mainly USA) cultures (6). The authors identified the four most common foci for the evaluation of clinical educators that comprised (in order of frequency): teacher (i.e., mapping onto the *information provider* in Harden and Crosby's work) and supporter (i.e., Harden and Crosby's *mentoring* role); role model; and feedback provider (i.e., relating to aspects of Harden and Crosby's *assessor* role). Planner and assessor roles were less common across intruments/items (featuring in 18 and 5 instruments respectively). The majority of manuscripts cited literature around the concept of good clinical teaching, but no clear description of what makes a good clinical educator is offered. Furthermore, the authors highlight that doctors' competencies “as communicators, collaborators, health advocates, and managers” are missing elements across these evaluation tools (6). More recently, however, within the health professions education literature there has been an attempt to develop clinical educator evaluation tools that variously cover aspects such as: teaching skills (7, 8), establishing learning climates (9), curriculum planning (10), communicating with learners (9), providing feedback (7, 8), assessing learners (9, 10), promoting self-directed learning (7, 10), demonstrating educational goals (10), showing responsibility (10), integrating learning into the clinical setting (9, 10), personal support (7). Many of the above abilities have been showed in the medical competencies described by the Canadian Medical Educational Directives (CanMEDS). It is reasonable that good clinical educators may be expected to act as role models of these competencies (6, 8).

### Who Are Best Placed to Evaluate Clinical Educators in Medical Education?

In their review of the literature, Fluit et al. (6) identified medical students as the most common raters of clinical educators (56% of the time). Indeed, some tools have been specifically developed for this purpose (11–13). It is easy to understand how medical students can be thought of as being the most suitable candidates for this task given that they are the recipients of education, and can observe the process directly. However, the power relationship between students and educators may interfere with the evaluation process. The crux of the issue is that educators have the power to fail students. As such, students may be reluctant to offend their educators as it might put them at risk of failure (14–18).

In addition to students, program coordinators also commonly evaluate clinical educators (19). While it is reasonable that program coordinators might score the clinical educators they assign, they may not have many opportunities to observe clinical educators' performances directly. In addition to students and program coordinators, clinical educators have been evaluated by their peers (2, 20, 21). Fellow educators may be able to observe one another during clinical teaching, or gather feedback about other educators from students. Finally, self-evaluation, a reflective process, has also been considered in several educator evaluation studies (2, 20–22).

Irrespective of whom is being evaluated and who undertakes the evaluation, the issue of the specific culture in which evaluation occurs is of importance. Here we focus on national culture (rather than local or organizational cultures). In East Asian cultures, such as Taiwan, China, Hong Kong, and Singapore, students are brought up to respect education and their educators (23–26). This impacts not only students' evaluation of their clinical educators, but also the evaluation of educators by others who are not afforded such reverence (e.g., program coordinators). As such this reverence for educators in general is likely to have an impact on if and how students, and other groups, might evaluate them. Furthermore, in certain contexts and cultures, competition between clinical educators may create a conflict of interest situation (27). Indeed, the literature on clinical educators' evaluation in the medical domain so far appears to come predominately from a Western perspective, with educators' accountability to students having been high on the agenda for a number of years, leading to a variety of evaluation contexts and processes (28–30). Given the different foci between an East Asian perspective and a Western perspective, an understanding of whether Taiwanese students' evaluation of their clinical educators differs from that of Western students would be of great interest; particularly due to the sharp rise in the internationalization of education in general (31) and healthcare professionals more specifically (32).

To date, research has studied evaluation systems available to examine the teaching quality of clinical educators (1, 7). However, there is no single, agreed source for this evaluation nor tool with which to evaluate all aspects of the educators' role: observing educators' effectiveness on different aspects of their role, and by a variety of raters, might be considered a more appropriate way to achieve an holistic view of performance (21, 33). Further, by integrating various subjective observations, we can begin to form a relatively objective evaluation of them. The aim of this study is to find out the suitable evaluation domains (*what*) and the appropriate raters (*who*) for evaluating clinical educators' competence in the context of medical education an East Asian culture: specifically Taiwan.

## Materials and Methods

### Study Setting

The study was conducted in the largest teaching hospital in Taiwan: a 3,000-bed Medical Center in the north of the country. There were approximately 920 clinical educators, 600 residents, 130 post graduate year physicians (PGY), 200 year-7 medical students (M7), 150 year-4, year-5, and year-6 medical students (M4–6), and at least 3,000 nurses in this institution during the study period.

### Ethical Approval

This study was approved by the Chang Gung Medical Foundation Institutional Review Board (REF: 103-1928B, anonymised to protect participants). Participants consented and were notified that they had the right to withdraw from the study at any time. They were also informed that they could omit any questions they did not want to respond to without consequences to their studies or work.

### Participants

A purposive sampling technique was used with the numbers of invitations being stratified by staffing ratios at the hospital. We invited a total of 500 particiants by sending questionnaires to 184 clinical educators, 120 residents, 26 PGYs, 40 M7, 30 M4–6, and 100 nurses at the study institution: All of whom should have had experiences of providing, witbessing or receiving clincal education in a hospital setting. The participants were invited by e-mail and were requested to complete the questionnaire online.

### Research Design

A quantitative cross-sectional survey design was used. The research questions (RQs) of this study were:

(RQ1) Which evaluation domains for the role of clinical educator do different stakeholder groups prioritize?
(RQ2) Which study population(s) do different stakeholder groups identify as being most suitable for the evaluation of clinical educators?

### Questionnaire Development

Drawing on our review of the literature (including: 1, 4, 5, 14, 21, 34–36), and our understanding of the educational environment of the institution, we itemized potential raters (i.e., *who*) and potential domains (i.e., *what*) for evaluating clinical educators. Five healthcare professional experts, including three physician educators, one nursing educator, and one questionnaire development expert at the study institution participated in a Delphi procedure (37, 38) to refine and validate the contents for the questionnaire. They exchanged their ideas under the condition of complete anonymity with the e-mail organized by the research assisstant. Ultimately, with the four rounds of written discussion, this process resulted in an 84-item web-based questionnaire with which we used to gather participants' opinions.

The questionnaire was divided into two parts. The first part, comprising 70 items and five domains focused on *what* we need to evaluate: teaching ability (16 items) (34, 39–43), assessment ability (12 items) (44–46), personal qualities (16 items) (4, 5, 40, 43, 47, 48), interpersonal relationships (14 items) (1, 2, 36, 43), and curriculum planning (12 items) (1, 22, 43, 49). The participants were asked to give a score to the question “*this item is suitable to be evaluated*” according to each domain. The second part, comprised 10 items, and focused on *who* is best placed to be the rater. The following possible rater groups, identified by evaluation experts in the study institution, were: clinical educator self-evaluation, clinical educators' peers, PGYs, residents, medical students, nurses, education associated administrative staff, outpatient services staff, and the clinical educators' supervisors. In this section, participants were asked to give a score for each of the potential rater group that is best placed to evaluate the clinical educators. To avoid a neutral option for respondents, both parts used the same 4-point Likert scale (1 = strongly disagree, 2 = disagree, 3 = agree, 4 = strongly agree). The anonymous questionnaire collected participants' age, gender, occupation, learning and teaching experience in years.

### Data Analysis

The quantitative responses were analyzed using SPSS 19.0 for Windows software package. For the descriptive statistics, we present the means ± standard deviations. Comparisons among the different groups were analyzed *via* a one-way analysis of variance (ANOVA) using the Scheffe test for *post hoc* analysis (*p* < 0.05).

## Results

Two hundred and fifty-eight individuals completed questionnaires (response rate 52%). They comprised mainly male participants (*n* = 177, 69%) and relatively fewer female participants (*n* = 81, 31%), with the mean ± SD of age: 28 ± 7 years. The breakdown by group was: clinical educators (*n* = 63, 24%), residents (*n* = 87, 34%), PGYs (*n* = 20, 8%), M7 (*n* = 31, 12%), M4–6 (*n* = 26, 10%), and nurses (*n* = 31, 12%). The M4–6 group had the highest response rate (87%), however, the nurse group had the lowest response rate (31%: Table 1).

**Table 1:** Demographic information of respondents.

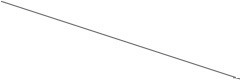	**Clinical educators**	**Residents**	**PGYs**	**M7**	**M4-6**	**Nurses**
#*n* (#*n* invited)	63 (184)	87 (120)	20 (26)	31 (40)	26 (30)	31 (100)
Response rate (%)	34	72	77	78	87	31
Gender (*M*%)	87	76	65	74	77	0
Age (mean ± SD)	37 ± 12	29 ± 5	27 ± 2	26 ± 2	22 ± 1	37 ± 7

### Which Evaluation Domains Are Prioritized?

All respondent groups, except nurses, agreed that *teaching ability* was the most important domain. We applied ANOVA test to evaluate the scores of *teaching ability* from the different groups showed a significant difference [F (92) = 0.838, *p* = *0.004*]. The *post hoc* Scheffe test showed a significant difference between the clinical educator and the nurse groups for *teaching ability* (*p* = *0.016*). The clinical educator, resident, M7 and M4–6 groups chose *assessment ability* as the least important domain. The M7 and M4–6 groups prioritized the five domains in an identical pattern to each other, with the clinical educator and resident groups prioritizing three of the five domains identically. The PGY group placed *curriculum planning* as the least important domain, although this was placed as the most important for the nurse group (Table 2).

**Table 2:** Mean±SD/Rank of the domains for evaluating clinical educators across different respondent groups.

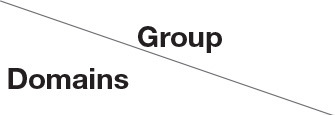	**Clinical educators**	**Residents**	**PGYs**	**M7**	**M4–6**	**Nurses**	***p* value of ANOVA test**
Teaching ability	3.38 ± 0.91/1*	3.23 ± 0.96 /1	3.26 ± 0.7 /1	3.28 ± 0.95 /1	3.28 ± 0.59 /1	2.72 ± 1.62 /5*	0.004
Assessment ability	2.97 ± 0.40/5	2.93 ± 0.70/5	2.83 ± 0.41 /4	2.80 ± 0.75 /5	2.78 ± 0.52 /5	3.25 ± 0.60 /2	0.060
Personal qualities	3.21 ± 0.53/2	3.11 ± 0.60 /2	3.02 ± 0.48 /3	3.15 ± 0.55 /3	3.05 ± 0.47 /3	3.21 ± 0.56 /3	0.144
Interpersonal relationship	3.08 ± 1.08/3	2.96 ± 1.11 /4	3.24 ± 1.03 /2	3.19 ± 1.14 /2	3.10 ± 0.55 /2	2.97 ± 1.48 /4	0.496
Curriculum planning	3.06 ± 0.46/4	2.97 ± 0.61 /3	2.76 ± 0.65 /5	2.89 ± 0.63 /4	2.90 ± 0.49 /4	3.39 ± 0.57 /1	0.061

*Scheffe test, clinical educator vs. nurses p = 0.016.

We undertook an in-depth comparison between the clinical educator and nurse groups due to the stark differences in their initial ranking of domains. When comparing the top five ranked items across all domains for these two groups, we can see that both groups of respondents selected identical items as their number one priority across all domains (Table 3). Additionally, for the domain of *personal qualities*, both groups selected the same top five items, albeit in a slightly different order. For the domains of *teaching ability* and *interpersonal relationships*, both groups prioritized 4/5 identical items in their top five: interestingly, in terms of *interpersonal relationships*, respondents from the nursing group only prioritized *demonstrates good interprofessionalism*, whereas respondents from the clinical educator group only prioritized *demonstrates mutual respect* (between themselves and learners). For the domain of *assessment ability*, the nurse group prioritized *uses assessment techniques well* and *good at formative assessment*, whereas the clinical educator group focused on *demonstrates good assessemt for clinical skills* and *clinical reasoning*. For the domain of *curriculum planning*, the nurse group prioritized *applies teaching resources effectively* and *demonstrates good curriculum management*, whereas the clinical educator group focused more on *demonstrates good time management* and *educates in accordance with the training program and schedule*.

**Table 3:** Comparison of the top 5 items prioritized by clinical educator and nurse groups across all domains.

**Domains**	**Priority**	**Clinical educators**	**Nurses**
Personal qualities	1	Enthusiasm 3.64 ± 0.64	Enthusiasm 3.76 ± 0.44
	2	Takes responsibility 3.64 ± 0.57	Mentors learners with patience 3.72 ± 0.45
	3	Demonstrates empathy 3.56 ± 0.65	Takes responsibility 3.69 ± 0.54
	4	Demonstrates leadership 3.52 ± 0.71	Demonstrates empathy 3.69 ± 0.47
	5	Mentors learners with patience 3.52 ± 0.59	Demonstrates leadership 3.69 ± 0.46
Teaching ability	1	The content of teaching 3.76 ± 0.44	The content of teaching 3.69 ± 0.47
	2	Guides clinical reasoning correctly 3.68 ± 0.56	Has ability to teach clinical skills 3.62 ± 0.49
	3	Has high level of teaching skills 3.68 ± 0.48	Guides clinical reasoning correctly 3.62 ± 0.47
	4	Has ability to teach clinical skills 3.56 ± 0.71	Promotes understanding and remembering 3.59 ± 0.57
	5	Promotes understanding and remembering 3.56 ± 0.65	Has good presentation skills 3.59 ± 0.50
Interpersonal relationship	1	Maintains patient privacy 3.60 ± 0.58	Maintains patient privacy 3.72 ± 0.53
	2	Facilitates good teacher-student interaction 3.60 ± 0.50	Demonstrates good patient-doctor interaction 3.69 ± 0.47
	3	Demonstrates mutual respect 3.56 ± 0.58	Demonstrates good communication skills 3.69 ± 0.46
	4	Demonstrates good patient-doctor interaction 3.52 ± 0.65	Facilitates good teacher-student interaction 3.66 ± 0.48
	5	Demonstrates good communication skills 3.48 ± 0.65	Demonstrates good interprofessionalism 3.66 ± 0.47
Assessment ability	1	Identifies students in difficulty 3.44 ± 0.65	Identifies students in difficulty 3.66 ± 0.55
	2	Demonstrates good assessment for clinical skills 3.40 ± 0.58	Be fair and objective 3.66 ± 0.48
	3	Demonstrates good assessment for clinical reasoning 3.32 ± 0.56	Uses assessment techniques well 3.62 ± 0.49
	4	Be fair and objective 3.24 ± 0.66	Good at summative assessment 3.59 ± 0.57
	5	Good at summative assessment 3.20 ± 0.65	Good at formative assessment 3.59 ± 0.50
Curriculum planning	1	Integrates teaching into clinical practice 3.60 ± 0.58	Integrates teaching into clinical practice 3.59 ± 0.50
	2	Prepares well for teaching 3.48 ± 0.59	Applies teaching resources effectively 3.58 ± 0.45
	3	Facilitates good practice-based learning and improvement 3.36 ± 0.64	Demonstrates good curriculum management 3.55 ± 0.51
	4	Demonstrates good time management 3.32 ± 0.69	Facilitates good practice-based learning and improvement 3.48 ± 0.57
	5	Educates in accordance with the training program and schedule 3.32 ± 0.68	Prepares well for teaching 3.48 ± 0.51

*Shaded areas indicate where items in the top 5 differ. The mean ± SD showed under each item was calculated from the reply of the questionnaire with 4-point Likert scale (1 = strongly disagree, 2 = disagree, 3 = agree, 4 = strongly agree)*.

### Which Group (s) Are Best Placed for Evaluating Clinical Educators?

We now turn our attentions to the *who* question: namely which stakeholder group respondents identify as being the most appropriate to undertake evaluation of clinical educators. Our data illustrates that clinical educators' supervisors, clinical educators themselves (so, self-evaluation), clinical educators' peers, residents, PGYs, M7, M4–6, and nurses featured in the top 5 suitable rater sources for each participant group. All six participant groups identified self-evaluation by clinical educators and PGYs in the top five of suitable rater sources (Table 4).

**Table 4:** The top five stakeholder groups (of 10) rated by respondent groups for evaluating clinical educators.

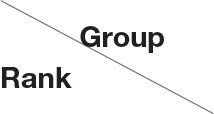	**Clinical educators**	**Residents**	**PGYs**	**M7**	**M4-6**	**Nurses**
1	Clinical educators' peers 3.52 ± 0.51	Residents 3.17 ± 0.86	PGY 3.19 ± 1.14	Residents 3.56 ± 0.81	PGY 3.43 ± 0.50	Clinical educators' self-evaluation 3.60 ± 0.50
2	Residents 3.51 ± 0.59	M7 3.13 ± 0.91	Residents 3.17 ± 1.17	PGY 3.50 ± 0.77	Residents 3.40 ± 0.54	M7 3.55 ± 0.60
3	PGY 3.48 ± 0.51	PGY 3.13 ± 0.89	M7 3.17 ± 1.14	M7 3.50 ± 0.77	Clinical educators' self-evaluation 3.36 ± 0.69	Clinical educators' self-evaluation 3.50 ± 0.51
4	M7 3.40 ± 0.50	Clinical educators' self-evaluation 3.10 ± 0.84	Clinical educators' self-evaluation 3.06 ± 1.13	M4-6 3.39 ± 0.80	M7 3.31 ± 0.56	PGY 3.50 ± 0.90
5	Clinical educators' self-evaluation 3.32 ± 0.75	M4-6 3.01 ± 0.88	M4-6 2.91 ± 0.13	Clinical educators' self-evaluation 3.36 ± 0.83	Clinical educators' self-evaluation 3.29 ± 0.56	Nurses 3.40 ± 0.99

## Discussion

This study investigated the suitable content and raters for doctors in their roles as clinical educators. In terms of content, Fluit et al. (6) identified *role model, teacher, feedback provider*, and *supporter* being the most common aspects of existing tools, with *assessor* and *planner* roles being less common. In our study, we identified the roles of *assessor* and *planner* as key factors. With the current international movement toward competency-based training programmes, and East Asia being no exception, the ability to assess the development of students' and trainees' knowledge, skills and practice is becoming a crucial component of the clinical educators' role. Clinical educators' abilities to plan learning activities in busy clinical environments, creating and protecting occasions for the execution of relevant clinical activities, provides a necessary structure for educators and learners. As such the planning role is important for clinical educators, and one for which they can be evaluated.

We found that clinical educators and medical learners prioritize educators' skills over other aspects of the clinical teaching role, including their personal qualities (e.g., enthusiasm, leadership, empathy), which were often ranked below interpersonal relationships by the learners. Indeed, both the clinical educators and medical learners in this study paid attention to the “learning process” and the “learning outcome” when thinking about eudcator evaluation. This contrasts with studies from Western cultural contexts which has found that clinical educators as role models, in which personal aspects such as leadership and enthusiasm are central components (50–52), and is more compatible with the results of studies that focus on the effectiveness of teaching (20, 35).

Our findings show that both clinical educators and learners ranked the five domains in a similar order: *teaching ability* rated as highest priority, *assessment ability* as the lowest (or very low). However, we believe that the rationale for each groups' ranking might be different. Thus, for the educators, their low ranking of assessment ability might be due to the relatively low confidence that clinical educators have in their assessment role (53). Indeed, clinical educators sometimes pass underperformance in their students (known as *failing to fail*): a difficult process tied up with educators' beliefs around self-efficacy (i.e., whether they have the ability to fail the student), their own knowledge and skills (e.g., whether they taught the student correct information, and in an appropriate way) as well as organizational constraints (the consequences of failing students on the organization, the program or fellow educators (53–56). Furthermore, the process of assessment and feedback can sometimes have a negative emotional impact on the educator (15). As such, these aspects of assessment behaviors might lead clinical educators to rate this domain as the lowest priority. As for students, their rationale for rating assessment ability as the lowest priority may be linked to assessment anxiety. This is supported in a recent systematic review examining assessment and psychological distress among medical students, which found that irrespective of the type of assessment involved or the level of learner, overwhelmingly assessment invokes stress or anxiety (57). As such, we might expect that students would display a reluctance to prioritize this aspect of their teachers' role.

In Stein et al.'s study (20) examining teaching effectiveness, different health professions prioritized different aspects of effective teaching according to their own professional culture. We also see this patterning whereby respondents in the nursing group ranked the clinical educator domains differently to respondents in the medical educator or learner groups, most notably for the domains of curriculum planning and assessment ability and teaching ability: which they ranked #1, #2, and #5 respectively, and which others ranked #3–5, #4–5, and #1 respectively. This stark difference in ranking might reflect differences in professional culture and focus alongside professional distance. Thus, in the Taiwanese culture in which this study was conducted, nurse education is highly prioritized and nurse education research comprises a high degree of teaching and curricula innovation evaluations (58–61). This may potentially explain why nurses prioritized the *curriculum planning* as their number-one domain. That nurses prioritize assessment more than do clinical educators and learners is likely to be due to the fact that they are far removed from the direct assessor-assessee relationship (62–64), and when assessing the learners they do so as part of a group and so the sole responsibility of passing or failing them does not primarily fall on their shoulders.

We undertook an additional comparison between the clinical educator and nurse groups based on their stark differences in opinions around the importance domains for clinical educator evaluations. We found that both groups selected *enthusiasm* as their #1 item across all the *personal qualities* domain, in addition to selecting the same top-5 for the domain of *personal qualities*. This suggests that there are more similarities than there are differences between the two groups. Our finding is compatible with several previous studies for both physicians (2, 22) and nurses (43) which showed that *enthusiasm* was identified as an important parameter of an effective teacher.

Where differences occurred between clinical educator and nurse groups, this was mainly in the domains of *assessment abilities* and *curriculum planning;* furthermore, only the nurse group prioritized the item *demonstrates good interprofessionalism* within the domain of *interpersonal relationships*. This latter finding around the respective prioritizing of interprofessional cooperation echoes research undertaken in the undergraduate domain that has found medical students hold more negative attitudes toward interprofessional communication and collaboration, and this holds over time despite interprofessional learning experiences (65–67). Furthermore, in everyday clinical practice and workplace learning, issues of interprofessional hierarchies, roles and conflicts come into play, leading to healthcare practitioners and learners experiencing interprofessional dilemmas (68): notably, due to the power asymmetry between nurses and doctors, it is the nurses who tend to experience greater distress as a result. It is therefore unsurprising that nurses might prioritize *interprofessionalism*.

Finally, our data showed that the self-evaluation and PGYs were the two most common sources identified for the evaluation of clinical educators. The importance of self-evaluation for the clinical educator evaluation has also been identified in previous studies (2, 20, 22). The Johari window (69), a technique that helps people understand relationships between themselves and others, highlights the importance of self-evaluation. The triangulation of evaluation including learners, peers, and self-evaluation (10, 65) should be considered in the design of future clinical educator evaluation.

As with any study, there are some limitations. Our study was conducted at a single site, with a relatively low response rate in comparison to the entire population targetted and inconsistentcy regarding to the different rater sources. Indeed, we had relatively high response rates from medical learners—including residents, PGYs and medical students—with the lowest response rates from clinical educators and nurses. Given that learners are on the receiving end of clinical educators' teaching, and are presently the main stakeholder group undertaking clinical educators' evaluation, it might be that they are more motivated to respond to invitations to complete the questionnaire about their clinical educators' evaluation. On the other hand, in East Asian cultures, educators comprise an occupation with a relatively high social status. In contrast to the learner-centered education, the educator-centered education may still exist in the East Asian cultures (14). It might be, therefore, that the relatively low response rate from the educators themselves is due to their unwillingness to be evaluated. Furthermore, due to the male-dominated composition of the clinical educators and medical students in Taiwan, our study participants comprised around 70% male. Thus, taken together, caution should be used when considering how the findings might be extrapolated to other settings.

## Conclusion

The evaluation of clinical educators is a critical, complicated, and difficult task. The purposes of educator evaluations are to provide learning opportunities for educators, to evaluate how educators able assist their students' learning, to help educators to enhance teaching abilities, and to reward excellent educators. The final goal of educator evaluation is to improve education effectiveness. In this study, we applied a questionnaire survey to find out the suitable evaluation domains/aspects and rater sources for the evaluation of clinical educators. We found that most participants listed *teaching ability* as their first priority, which was different to the research findings from Western contexts. In terms of the evaluation aspects in each domain, the study showed that the rater sources have both similar and different choices. This reflects the rater sources' opinions of the evaluation of clinical educators according to their experiences. Regarding the issue of *who* is best placed to assess clinical educators, the study showed the best fit rater groups were educators themselves and PGYs. This study gathered the information about the similar and different opinions from different sources in a single East Asian context. Further studies may be drawn upon for the creation of a comprehensive evaluation system for clinical educators in other East Asian contexts according to the results of this study.

## Data Availability Statement

The raw data supporting the conclusions of this article will be made available by the authors, without undue reservation.

## Ethics Statement

The studies involving human participants were reviewed and approved by Chang Gung Medical Foundation Institutional Review Board. Written informed consent for participation was not required for this study in accordance with the national legislation and the institutional requirements.

## Author Contributions

C-CJ: design of the work, drafting, and revising. L-SO: analysis and interpretation of data. H-MT: analysis and interpretation of data and revising. Y-PC: collecting data. J-RL: analyzing data. LM: analysis and interpretation data, revising, and final approval for publishing. All authors contributed to the article and approved the submitted version.

## Funding

This study was supported by grants from the Chang Gung Medical Research Fund (CDRPG3D0021), Department of Medicine of Chang Gung Memorial Hospital, and Chang Gung Medical Education Research Center (CGMERC).

## Conflict of Interest

The authors declare that the research was conducted in the absence of any commercial or financial relationships that could be construed as a potential conflict of interest.

## Publisher's Note

All claims expressed in this article are solely those of the authors and do not necessarily represent those of their affiliated organizations, or those of the publisher, the editors and the reviewers. Any product that may be evaluated in this article, or claim that may be made by its manufacturer, is not guaranteed or endorsed by the publisher.
