# Supplementary material for: Evaluating Clinical Educators' Competence in an East Asian Context: Who Values What?
Source: Front Med (Lausanne). 2022 Jun 28;9:896822. doi: 10.3389/fmed.2022.896822 (PMC9273768; doi:10.3389/fmed.2022.896822)
Supplement: Supplementary file 1 [file Data_Sheet_1.PDF]

The basic personal information

Please fill in the following information in accordance with the state when you fill in this questionnaire.

1. Age: _____ years old
2. Gender: ☐Male ☐Female
3. Role:

☐ Medical Students (☐ Fourth Grade ☐ Fifth Grade ☐ Sixth Grade ☐ Seventh Grade)

☐ PGY

☐ Residency

Division: ☐ Internal medicine ☐Surgery ☐Pediatric ☐Obstetrics Gynecology ☐Emergency

☐ Other __________

Seniority: ☐ First Year ☐Second Year ☐Third Year ☐Fourth Year ☐Fifth Year ☐ Sixth Year

☐ Nurses, with teaching experiences _____ years

☐ Clinical educators, with teaching experiences _____ years

Division: ☐ Internal medicine ☐Surgery ☐Pediatric ☐Obstetrics Gynecology ☐Emergency

☐ Other __________

<u>**The first part**</u>

The first part of this questionnaire is to ask about the suitable domains and items of the clinical educator's evaluation. According to the literature review, we have listed the five main domains for evaluating clinical educators in medicine, and the items belong to each domain in the following tables. Please circle an appropriate number from 1-4 to present your opinions based on the views of evaluating clinical educators.

■ The "**teaching ability**" is suitable domain during evaluating the clinical educators.

**Domain**	**Strongly Disagree**	**Disagree**	**Agree**	**Strongly Agree**
**Teaching ability**	1	2	3	4

■ The following items belong to the domain "**teaching ability**" are suitable items during evaluating the clinical educators.

**Item**	**Strongly Disagree**	**Disagree**	**Agree**	**Strongly Agree**
1. Has high level of teaching skills	1	2	3	4
2. Professional knowledge	1	2	3	4
3. The content of teaching	1	2	3	4
4. The use of evidence-based medicine approach	1	2	3	4
5. Has ability to teach clinical skills	1	2	3	4
6. Has good presentation skills	1	2	3	4
7. Produces good teaching material	1	2	3	4
8. Multimedia applications	1	2	3	4
9. Promotes understanding and remembering	1	2	3	4
10. Guides clinical reasoning correctly	1	2	3	4
11. Demonstrates good small group teaching skills	1	2	3	4
12. Demonstrates good large group teaching skills	1	2	3	4
13. Demonstrates good lecture skills	1	2	3	4
14. Demonstrates good one-to-one teaching skills	1	2	3	4
15. Demonstrates teaching innovation	1	2	3	4
16. Facilitates problem-oriented learning	1	2	3	4

■ The "**assessment ability**" is suitable domain during evaluating the clinical educators.

Domain	Strongly Disagree	Disagree	Agree	Strongly Agree
**Assessment ability**	1	2	3	4

■ The following items belong to the domain "**assessment ability**" are suitable items during evaluating the clinical educators.

Item	Strongly Disagree	Disagree	Agree	Strongly Agree
1. Assessment techniques	1	2	3	4
2. Be fair and objective	1	2	3	4
3. Uses assessment techniques well	1	2	3	4
4. Out of question written test capability	1	2	3	4
5. Identifies students in difficulty	1	2	3	4
6. Good at formative assessment	1	2	3	4
7. Good at summative assessment	1	2	3	4
8. Records for evaluation of guidance	1	2	3	4
9. Demonstrates good assessment for clinical reasoning	1	2	3	4
10. Demonstrates good assessment for clinical skills	1	2	3	4
11. Understand learner	1	2	3	4
12. Good use of assessment outcomes	1	2	3	4

■ The "**personal qualities**" is suitable domain during evaluating the clinical educators.

Domain	Strongly Disagree	Disagree	Agree	Strongly Agree
**Personal qualities**	1	2	3	4

■ The following items belong to the domain "**personal qualities**" are suitable items during evaluating the clinical educators.

Item	Strongly Disagree	Disagree	Agree	Strongly Agree
1. Enthusiasm	1	2	3	4
2. Takes responsibility	1	2	3	4
3. Acts as a role model	1	2	3	4
4. Promotes self-learning in students	1	2	3	4
5. Mentors learners with patience	1	2	3	4
6. Contributes towards behavior change and progress	1	2	3	4
7. Demonstrates a professional attitude	1	2	3	4
8. Compassionate	1	2	3	4
9. Demonstrate leadership	1	2	3	4
10. Demonstrates self-confidence	1	2	3	4
11. Demonstrates reflexivity	1	2	3	4
12. Be self-adapting	1	2	3	4
13. Demonstrate empathy	1	2	3	4
14. Dose not discriminate	1	2	3	4
15. Attracts learners	1	2	3	4
16. Demonstrates fallibility	1	2	3	4

■ The "**interpersonal relationship**" is suitable domain during evaluating the clinical educators.

Domain	Strongly Disagree	Disagree	Agree	Strongly Agree
**Interpersonal relationship**	1	2	3	4

■ The following items belong to the domain " **interpersonal relationship** " are suitable items during evaluating the clinical educators.

Item		Strongly Disagree	Disagree	Agree	Strongly Agree
1.	Interacts well with medical team	1	2	3	4
2.	Administrative cooperation	1	2	3	4
3.	Demonstrates good patient-doctor interaction	1	2	3	4
4.	Treats patients the way you treat your family	1	2	3	4
5.	Facilitates good teacher-student interaction	1	2	3	4
6.	Shared responsibility between teacher and learners	1	2	3	4
7.	Demonstrates good interprofessionalism	1	2	3	4
8.	Demonstrate good communication skills	1	2	3	4
9.	Support learners	1	2	3	4
10.	Maintains patient privacy	1	2	3	4
11.	Demonstrates mutual respect	1	2	3	4
12.	Be approachable and accessible	1	2	3	4
13.	Provides effective feedback	1	2	3	4
14.	Has a pleasant demeanour	1	2	3	4

■ The " **curriculum planning** " is suitable domain during evaluating the clinical educators.

Domain	Strongly Disagree	Disagree	Agree	Strongly Agree
**Curriculum planning**	1	2	3	4

■ The following items belong to the domain " **curriculum planning** " are suitable items during evaluating the clinical educators.

Item	Strongly Disagree	Disagree	Agree	Strongly Agree
1. Demonstrates good teaching organisation	1	2	3	4
2. Integrates teaching into clinical practice	1	2	3	4
3. Facilitates good practice-based learning and improvement	1	2	3	4
4. Demonstrates good system-based practice	1	2	3	4
5. Applies teaching resources effectively	1	2	3	4
6. Constructs a positive learning atmosphere	1	2	3	4
7. Demonstrates good curriculum management	1	2	3	4
8. Demonstrates good time management	1	2	3	4
9. Prepares well for teaching	1	2	3	4
10. Be approachable for learners' assistance	1	2	3	4
11. Educates in accordance with the training program and schedule	1	2	3	4
12. Demonstrates willingness to improve teaching skills	1	2	3	4

The second part

The second part of this questionnaire is to ask about each of the following group is best placed to evaluate the clinical educators. Please circle an appropriate number from 1-4 to present your opinions based on the views of evaluating clinical educators.

Rater group		Strongly Disagree	Disagree	Agree	Strongly Agree
1.	Clinical educator self-evaluation	1	2	3	4
2.	Clinical educators' peers	1	2	3	4
3.	Post-graduate year medical trainers (PGYs)	1	2	3	4
4.	Residents	1	2	3	4
5.	Year-7 medical students (M7)	1	2	3	4
6.	Year-4~6 medical students (M4-6)	1	2	3	4
7.	Nurse	1	2	3	4
8.	Outpatient services staff	1	2	3	4
9.	Clinical educators' supervisors	1	2	3	4
10.	Medical education associated administrative staff	1	2	3	4

The entire questionnaire is over, thank you to fill!
